# European Code Against Cancer, 5th edition – organised cancer screening programmes

**DOI:** 10.1002/1878-0261.70197

**Published:** 2026-01-16

**Authors:** Esther Toes‐Zoutendijk, Marc Arbyn, Anssi Auvinen, David Baldwin, Xavier Castells, Andrea DeCensi, Solveig Hofvind, Urska Ivanus, Carlo Senore, Mangesh Thorat, Carlijn van der Aalst, Ana Carolina Pereira Nunes Pinto, Javier Bracchiglione, Ariadna Feliu, Hajo Zeeb, Erica D'Souza, David Ritchie, Carolina Espina, Andre L. Carvalho, Iris Lansdorp‐Vogelaar

**Affiliations:** ^1^ Public Health, Erasmus MC University Medical Centre Rotterdam Netherlands; ^2^ Unit of Cancer Epidemiology Belgian Cancer Centre, Sciensano Brussels Belgium; ^3^ Faculty of Social Sciences Tampere University and Tampere University Hospital Finland; ^4^ Nottingham University Hospitals, Respiratory Medicine Unit David Evans Centre, Nottingham City Hospital Campus UK; ^5^ Department of Epidemiology and Evaluation Hospital del Mar Research Institute Barcelona Spain; ^6^ EO Ospedali Galliera Genoa Italy; ^7^ Department of Breast Cancer Screening, Cancer Registry Norwegian Institute of Public Health Oslo Norway; ^8^ Department of Cervical Cancer Screening, Epidemiology and Cancer Registry Institute of Oncology Ljubljana Slovenia; ^9^ Epidemiology and screening unit – CPO University hospital Città della Salute e dela Scienza Turin Italy; ^10^ Centre for Cancer Screening, Prevention and Early Diagnosis, Wolfson Institute of Population Health Queen Mary University of London UK; ^11^ Breast Services Homerton University Hospital London UK; ^12^ Iberoamerican Cochrane Centre Institut de Recerca Sant Pau (IR Sant Pau) Barcelona Spain; ^13^ Institut de Recerca Sant Pau (IR SANT PAU) Barcelona Spain; ^14^ Interdisciplinary Centre for Health Studies (CIESAL) Universidad de Valparaíso Viña del Mar Chile; ^15^ Centro de Investigación Biomédica en Red de Epidemiología y Salud Pública Instituto de Salud Carlos III Madrid Spain; ^16^ International Agency for Research on Cancer (IARC/WHO) Lyon France; ^17^ Department of Primary Care and Public Health, School of Public Health Imperial College London UK; ^18^ Leibniz‐Institute for Prevention Research and Epidemiology‐BIPS, Bremen Germany; ^19^ Health Sciences, University of Bremen Germany

**Keywords:** breast cancer, cervical cancer, colorectal cancer, Europe, European Code Against Cancer, lung cancer, organised cancer screening, prostate

## Abstract

The 5th edition of the European Code Against Cancer (ECAC5) recommends sustainable, organised screening programmes for: (a) colorectal cancer using biennial quantitative faecal immunochemical test (FIT) for individuals aged 50–74 years. As an alternative strategy, once‐only endoscopy may be considered within the same age range; (b) breast cancer using biennial digital mammography for women aged 50–69 years. Implementing this strategy for women aged 45–49 years and 70–74 years can be considered. Other screening strategies or additional examinations could be considered for women with high mammographic density; (c) cervical cancer using human papillomavirus (HPV) screening at intervals no shorter than 5 years for women aged 30–65 years. It is recommended to adapt policies according to vaccination status and screening history; and (d) lung cancer using annual low‐dose computed tomography (LDCT) for individuals considered to be at increased risk of lung cancer based on age, history of smoking or validated risk models, with biennial screening as an alternative. Screening should incorporate smoking cessation interventions.

## Graphical abstract

ECAC5 updates cancer prevention recommendations on cancer screening. Individuals are recommended to take part in organised screening programmes for colorectal, breast, cervical and lung cancer, as recommended in their country. Policymakers should aim to implement sustainable, organised screening programmes for these cancer types to facilitate their population in participating in screening.

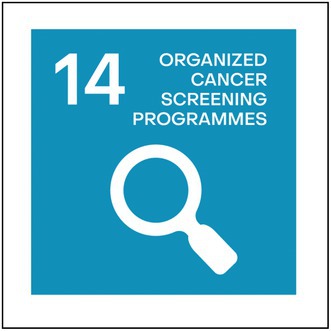

**Abbreviations**
CEAs: Cost‐effectiveness analyses
CIN3+: Cervical grade III intraepithelial neoplasia
COPD: Chronic obstructive pulmonary disease
ECAC: European Code Against Cancer
ECAC4: European Code Against Cancer, fourth edition
ECAC5: European Code Against Cancer, fifth edition
EU: European Union
FIT: Faecal Immunochemical Test
FS: Flexible sigmoidoscopy
gFOBT: GLuaiac‐based faecal occult blood testing
HPV: Human papillomavirus
IARC: International Agency for Research on Cancer
LDCT: Low‐dose computed tomography
MRI: Magnetic resonance imaging
PSA: Prostate‐specific antigen
QALY: Quality adjusted life year
RCT: Randomised controlled trials

## 1 Introduction

Europe accounts for 22% of the worldwide cancer incidence and 20% of cancer mortality, despite having only 10% of the world's population [1]. In Europe, prostate cancer was the most common cancer diagnosed in men in 2022, with an age‐standardised incidence rate of 59.9 per 100 000 males, while breast cancer was the most common cancer diagnosed in women (75.6 per 100 000 females). Colorectal cancer was the second most common cancer in both sexes (30.5 per 100 000 persons), followed by lung cancer (28.8 per 100 00 persons). Cervical cancer ranked 10th in women (10.6 per 100 000 females). Together, these five cancers accounted for about 47% of the cancer incidence in Europe. Lung cancer is the most common cause of cancer‐related death in Europe, with an age‐standardised mortality rate of 21.4 per 100 000 persons, followed by colorectal cancer (12.1 per 100 000 persons). Prostate cancer is third in men (11.2 per 100 000 males), breast cancer is first (14.6 per 100 000 females) and cervical cancer is 10th in women (3.9 per 100 000 females). For both colorectal cancer and lung cancer, the incidence rates are higher in men than in women. Most of the incidence and mortality patterns are characterised by a substantial socio‐economic gradient, with generally increasing incidence in low‐ and middle‐income countries and a decreasing incidence in high‐income countries. As a result, there are differences in incidence and mortality within Europe. Mortality rates for breast cancer were lower in Northern and Western Europe due to high screening coverage and accessibility to improved treatment. For cervical cancer, a clear east–west gradient is observed; the mortality rate in Eastern Europe of 6.3 per 100 000 females versus 3.9 per 100 000 females in the rest of Europe. Incidence trends typically dropped since the last decades of the previous century up to the earliest years of the current century, which paralleled the spread of mass screening in Western Europe and the Nordic countries [2]. Since then, several European countries with traditionally well‐organised cytology‐based screening programmes have shown stable or even increasing trends in cervical cancer incidence. In contrast, countries where screening coverage and the quality of cytological examination of Pap smears were poor to moderate at the end of the 1990s have observed declining incidence rates following the introduction of organised screening in the early 21st century. Differences in incidence rates were also observed for lung cancer, with higher rates in men in Eastern Europe and in women in Northern Europe [1], reflecting the course of the tobacco epidemic [3].

**Fig. 1:**
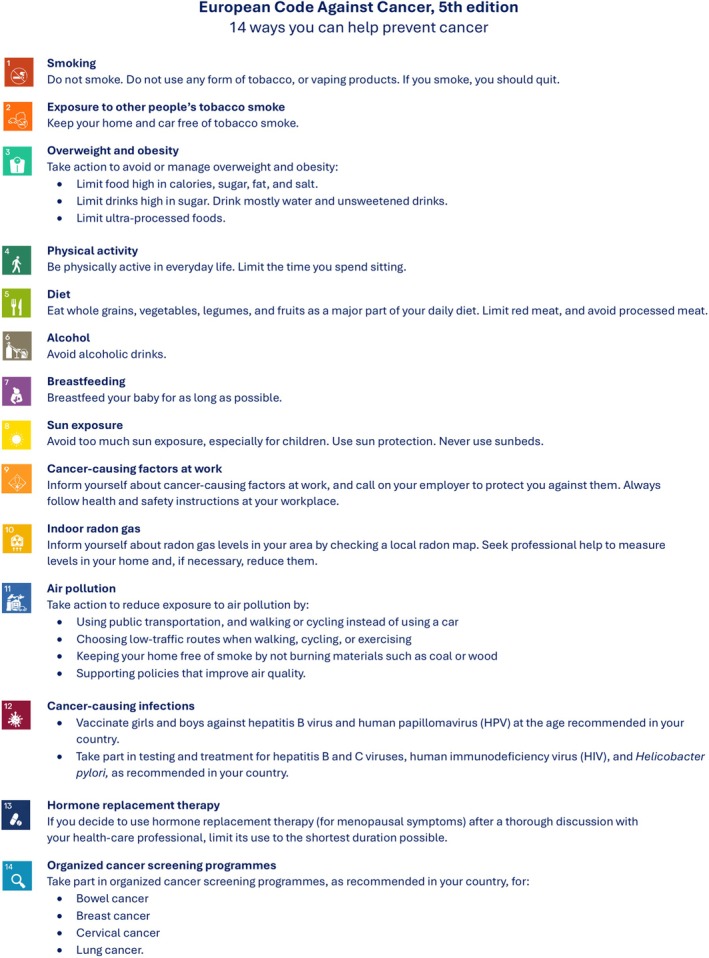
European Code Against Cancer, 5th edition: recommendations for individuals. The 14 recommendations of the European Code Against Cancer, 5th edition (ECAC5) adopted by the Scientific Committee of the ECAC5 project. © 2026 International Agency for Research on Cancer / WHO. Used with permission.

The high burden of cancer in Europe can be reduced by implementing evidence‐based screening programmes, alongside other preventive measures such as smoking cessation and vaccination. The Council of the European Union (EU) recommended organised screening for colorectal, breast and cervical cancer in 2003 [4]. Since 2022, the EU also recommends that the feasibility and effectiveness of lung cancer, prostate cancer and screen‐and‐treat strategies for *Helicobacter pylori* to reduce gastric cancer should be explored [4]. An overview of 28 European countries showed that colorectal, breast and cervical cancer screening programmes have been widely implemented in Europe: 25 countries for colorectal cancer, 23 for breast cancer and 24 for cervical cancer [5]. Moreover, the evidence from randomised controlled trials (RCTs), together with the recommendations of the EU Council, have led to several initiatives to evaluate the feasibility and effectiveness of organised lung and prostate cancer screening programmes in Europe [6]. This fifth edition of the European Code Against Cancer (ECAC5) evaluates the latest evidence on screening for all cancers recommended for screening by the EU Council, to update the ECAC4 cancer screening recommendations (Fig. 1, Annex S1) [7]. This paper presents the updated ECAC5 cancer screening recommendation for the public and the new cancer screening recommendation for policymakers, together with a summary of the supporting evidence. The evidence on gastric cancer screening has been performed by the Working Group on infections [8].

## 2 Approach

ECAC is an initiative of the European Commission designed to provide clear, evidence‐based recommendations for cancer prevention accessible to the public. The current 5th edition has been coordinated by the International Agency for Research on Cancer (IARC) as part of the World Code Against Cancer Framework, launched by IARC in 2022 [9]. The aim of the framework is to support the development of region‐specific Codes Against Cancer tailored to distinct epidemiological and socio‐economic contexts [9]. A specific methodology has been constructed for use in the development of any Regional Code, including ECAC5, as described in the methodology paper [10]. For the first time, ECAC5 is aimed not only at individuals, but also at policymakers (see Annex S1 for the complete ECAC5 recommendation for individuals and policymakers).

As a general principle, when evaluating the evidence to support a recommendation, the current scientific body of evidence should be classified as ‘sufficient’. This classification should come from authoritative sources, such as the IARC handbooks of cancer prevention, as described by Espina et al. [10]. When no such classification was available for a particular cancer type, a systematic literature review or synthesis of reviews was performed to assess the evidence. To be recommended, the available evidence must demonstrate that screening leads to a reduction in cancer incidence and/or mortality. Furthermore, the evidence should show that the benefits of adopting the recommendation outweigh the potential harms according to the judgement of the Working Group experts. The effectiveness of colorectal, breast and cervical cancer screening was thoroughly reviewed in ECAC4. Since then, no evidence has emerged to substantiate or challenge that conclusion, and we shortly summarise the evidence and relevant updates in the subsequent paragraphs. For lung and prostate cancer screening, the available evidence was not assessed in ECAC4, prompting a formal systematic review for each and thus the evidence is described in more detail. The PICOD criteria for the reviews are presented in Annex S2. The review for lung cancer screening was conducted as a synthesis of systematic reviews.

If evidence of effectiveness of screening for the specific cancer type was deemed sufficient, and the balance between harms and benefits of screening was found to be favourable, the following dimensions were evaluated: equity, feasibility, and individual actionability. Screening for that cancer was then recommended if its impact was deemed to ensure a favourable impact on all these dimensions by the expert Working Group. For the recommended cancer screenings, the corresponding European and World Health Organisation (WHO) guidelines were reviewed, and policy recommendations were derived from these guidelines [10]. For the policy‐level recommendation, the recommended test, age range, feasibility and required resources were evaluated.

## 3 Recommendation for individuals

### 3.1 Scientific justification for inclusion and update of the recommendation in ECAC5

#### 3.1.1 Evidence on the effectiveness of cancer screening

A comprehensive evaluation of the effectiveness and assessment of benefits and harms associated with screening for each of the cancers is presented below. This evaluation considers the potential benefits—namely incidence and mortality reductions—and the potential harms—namely false‐positive results, overdiagnoses and complications—to assess whether the overall balance is favourable.

##### 3.1.1.1 Colorectal cancer screening (bowel cancer screening)

Meta‐analysis of RCTs of guaiac‐based faecal occult blood testing (gFOBT) showed a 12% reduction in colorectal cancer mortality (Relative Risk (RR) 0.88, 95% CI 0.78–0.90) [11]. Numerous studies have demonstrated that faecal immunochemical testing (FIT) is higher in sensitivity for colorectal cancer than gFOBT. When combined with its ability to achieve higher participation rates, it is considered the preferred screening method over gFOBT [12, 13, 14, 15, 16]. Flexible sigmoidoscopy (FS) screening showed a mortality reduction of 25% (HR 0.75, 95% CI 0.67–0.83) and incidence reduction of 24% (HR 0.76, 95% 0.72–0.81) [17]. Since ECAC4, an RCT evaluating the effectiveness of colonoscopy screening has been published, showing no significant reduction in mortality (RR 0.90, 95% CI 0.64–1.16), and a reduction in incidence of 18% (RR 0.82, 95% CI 0.70–0.93) [18]. The per‐protocol analyses did show a significant cause‐specific mortality reduction of 50% (RR 0.50, 95% CI 0.27–0.77) as well as an incidence reduction of 31% (RR 0.69. 95% CI 0.55–0.83) [18].

A potential harm associated with colorectal cancer screening is the psychological risk following a positive test result and fear of a cancer diagnosis [19, 20]. Especially a false‐positive result is regarded as a potential harm because of the possible distress associated with a positive FIT result and the potential complications of the unnecessary colonoscopy. A barrier specific for FIT screening is the necessity of handling stool, which can be considered unpleasant and embarrassing [21]. Overdiagnosis is not considered a concern for colorectal cancer screening, as the prevention of cancer through screening may offset the potential increase in detected cancers [22]. While FIT screening is not associated with major complications, individuals who test positive must undergo colonoscopy, which carries a risk of serious or even fatal complications [23]. Endoscopy screening is more invasive and carries a higher risk of adverse effects, as primary colonoscopy screening requires all eligible individuals to undergo colonoscopy, not just those at higher risk (i.e., positive FIT), thereby exposing more people to potential complications [23, 24]. Fatal complications after colonoscopy are relatively rare: 3 to 7 deaths per 100 000 colonoscopies [23, 25].

Based on the evidence, it was concluded that the benefits of colorectal cancer screening outweigh the harms.

##### 3.1.1.2 Breast cancer screening

Meta‐analyses of RCTs of biennial mammographic screening showed a statistically significant 18–23% reduction in breast cancer mortality [26]. In a systematic review that included observational studies evaluating population‐based programmes, the observed mortality reduction was between 20 and 28% in invited women and 31 and 58% in participating women [27]. The benefits were more pronounced for those who attended regularly. In terms of potential harms, mammographic screening might be associated with similar harms as for the other cancer screening programmes, such as fear of cancer diagnosis and false‐positive test results [27]. All participants are exposed to low doses of radiation from mammography. This is particularly concerning for individuals with false‐positive results, as it may contribute to the development of breast cancer and may result in additional deaths from breast cancer, although this is likely to be extremely low/negligible [28]. Lowering the starting age to 40 would significantly increase the radiation exposure [29]. The most debated issue related to breast cancer is overdiagnosis and overtreatment, largely due to the detection and treatment of slow‐growing cancers that would likely not have been diagnosed if the women had not participated in screening. A review of European observational studies from 2012 showed an overdiagnosis proportion of between 1 and 10% [30]. A barrier specific for breast cancer screening is the discomfort or pain associated with undergoing mammography examination, which can affect individuals' willingness to participate [27]. No major complications are associated with mammography screening.

This evidence led to the conclusion that mammographic screening for breast cancer is beneficial and outweighs the harms.

##### 3.1.1.3 Cervical cancer screening

As the practice of microscopic interpretation of Pap smear was widely utilised before RCTs becoming the standard for generating evidence, no RCTs on the effectiveness of cytology screening have been conducted [31]. A meta‐analysis of observational studies showed a 17–79% reduction in cervical cancer mortality for invited vs. noninvited women [32]. The mortality reduction for women who participate in cervical cancer screening compared to those who do not ranges from 41 to 92%. Beyond effects on mortality, cytology screening showed an incidence reduction of 60% [33, 34, 35, 36]. Meta‐analysis showed that HPV‐based screening tests have a relative sensitivity *of* 1.37 (95% CI 1.20–1.55) for detecting CIN3+ compared to cytology testing [31]. RCTs showed a pooled reduction in CIN3+ detection in the second screening round for women with a negative baseline HPV test (RR 0.43, 95% CI 0.33–0.56) compared to those with a negative cytology test [33]. After 5 years, invasive cervical cancer was reduced by 0.45 (95% CI 0.25–0.81) in the HPV arm compared to the cytology arm [34]. Furthermore, HPV testing using validated PCR‐based assays on self‐collected specimens is as accurate in detecting CIN3+ as testing on cervical specimens collected by health professionals [37].

There are specific negative consequences associated with cervical cancer screening [31]. The psychological stress associated with a positive HPV test is more pronounced than that associated with an abnormal cytology test, given that HPV is a sexually transmitted disease [38, 39]. This may give rise to feelings of stigma and shame [40]. It has been documented that women referred for colposcopy may experience pain or discomfort. Like with colorectal cancer screening, overdiagnosis is not a major concern in cervical cancer screening, as screening also effectively prevents cancer by identifying and treating precancerous lesions. Complications of the surgical treatment of cervical precancerous lesions may increase the risk of preterm delivery and other adverse pregnancy outcomes in subsequent pregnancies [41, 42].

Based on this evidence, it was concluded that the harms associated with cervical cancer screening are limited and outweighed by the benefits.

##### 3.1.1.4 Lung cancer screening

The review of the thirteen published systematic reviews was completed as part of the update of the ECAC 5th Edition (Annex S3) [43]. Only three systematic reviews were at low risk of bias and included in the analysis. These reported on the use of low‐dose CT screening (LDCT) in high‐risk populations, defined by a personal history of current or previous tobacco smoking in addition to specified age ranges and some other risk factors. The reviews showed that LDCT screening reduced lung cancer mortality by 21% (RR 0.79, 95% CI 0.72–0.87, 8 trials, 91 122 participants); all‐cause mortality was reduced by 5% (RR 0.95, 95% CI 0.91–0.99, 8 trials, 91 107 participants). The overall incidence of lung cancer up to 7 years after the screening was 17% higher than without screening (RR 1.17, 95% CI 1.02–1.33), due to an increase in early‐stage lung cancer diagnoses, while incidence of advanced‐stage lung cancer was decreased by 25% (RR 0.75, 95% CI 0.68–0.83). Subgroup analyses showed a reduction in lung cancer mortality of 29% (RR 0.71, 95% CI 0.59–0.86) for women and a smaller reduction of 15% (RR 0.85, 95% CI 0.76–0.95) for men.

There is evidence that lung cancer screening is beneficial for high‐risk populations, defined by a personal history of current or previous tobacco smoking in addition to specified age ranges and some other risk factors. Those who meet the eligibility criteria should take part in the screening programme, as advised by local authorities.

Potential harms of lung cancer screening include both physical and psychological impacts, mostly due to associated worry and anxiety about the test results. Although LDCT uses X‐rays, the dose of radiation is very low. After 20 annual screening CT scans, the additional risk of developing cancer would be 0.22% for women and 0.12% for men [44]. LDCT detects lung cancer, pulmonary nodules that may or may not be cancer, mostly only requiring surveillance LDCT to detect interval growth, and incidental findings in the thorax and upper abdomen. There are well‐established management paradigms for lung cancer and pulmonary nodules that aim to minimise harm and stress for the participant while ensuring a definitive and timely diagnosis and treatment [45, 46]. Incidental findings may require further testing and treatment, which can offer benefits but also pose potential harms, such as identifying conditions that do little harm or that do not have beneficial interventions [47]. A systematic review with meta‐analysis on overdiagnosis of LDCT screening was conducted within the ECAC5 project [48]. To summarise, the meta‐analysis incorporated the findings of eight RCTs and two cost‐effectiveness studies. For the comparison between LDCT versus no screening, a nonsignificant rate of 5% overdiagnoses (RR 1.05, 95% CI 0.88–1.25) was estimated, representing 222 additional cases per 100 000 individuals screened. When using chest X‐ray as the comparator, little to no increase in overdiagnoses was observed (RR 1.01, 95% CI 0.95–1.08), representing 63 additional cases per 100 000 people screened [49]. Other possible harms are related to complications of additional diagnostic investigations of suspicious lung nodules, such as haemothorax or infection.

Based on this evidence, it was concluded that the harms associated with lung cancer screening are outweighed by the benefits.

##### 3.1.1.5 Prostate cancer screening

RCTs performed during the last decade have shown that Prostate‐Specific Antigen (PSA) screening can reduce mortality by up to 20% with individual RCTs showing variable reductions, most likely due to contamination from opportunistic screening in the control group [50, 51]. The most significant harm of prostate cancer screening is overdiagnosis, which can reach as high as 40%, depending on PSA levels and Gleason scores [52, 53]. Prostate cancer screening is associated with biopsy‐ and treatment‐related complications such as sepsis, urinary continence or erectile dysfunction [50].

**Fig. 2:**
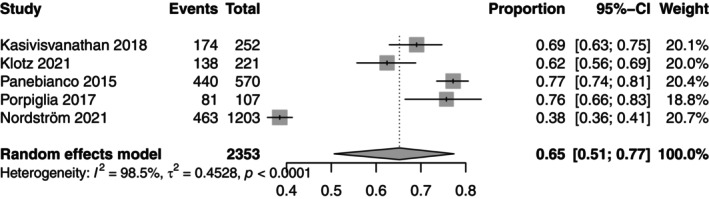
Forest plot biopsy frequency using MRI and MRI‐targeted biopsy among PSA positive men. Figure 2 presents meta‐analysis on biopsy frequency including five studies [55, 56, 57, 58, 59]. The square represents the point estimate of the individual study and the horizontal lines represent the 95% CI. The diamond represents the pooled effect estimate from all included studies.

The harms, especially overdiagnosis, outweigh the mortality reduction. However, harms could be reduced by using Magnetic Resonance Imaging (MRI)A pAscanning in men with abnormal PSA levels followed by MRI‐targeted biopsies if deemed necessary. The effectiveness of prostate cancer screening using MRI is unknown. A systematic review was conducted and nine RCTs—performed from 2015 to 2021 ‐were selected to be included in the meta‐analysis (November 2023) [54]. However, eight of the included studies represented diagnostic accuracy studies and only one concerned a screening trial. Figure 2 shows that biopsy frequency can significantly be reduced using MRI and MRI‐targeted biopsy (0.65, 95% CI 0.51–0.77), based on five studies with available data.

**Fig. 3:**
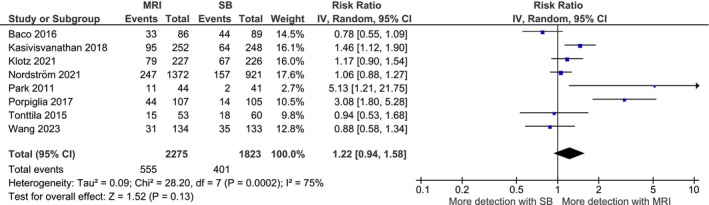
Forest plot for detection of clinically significant cancers. Figure 3 presents the meta‐analysis for clinically significant cancer detection including eight studies [55, 57, 58, 59, 60, 61, 62, 63]. The square represents the point estimate of the individual study and the horizontal lines the 95% CI. The diamond represents the pooled effect estimate of all included studies. MRI, magnetic resonance imaging; SB, systematic biopsy.

Eight studies were included to assess the impact on the detection of clinically significant cancers (Gleason score 7 or higher) (Fig. 3). MRI screening showed an increase in the detection of clinically significant cancers, but the magnitude of the increase was not statistically significant (RR 1.22, 95% CI 0.94–1.58). A sensitivity analysis excluding the study of Baco *et al*. (2016), because of a discrepancy in the outcome definition, marginally changed the initial results (RR 1.32, 95% CI 1.00–1.73). Importantly, the meta‐analysis showed that the use of MRI compared to standard care resulted in a significant reduction in the detection of clinically insignificant cancers (Gleason < 7) (RR 0.52, 95% CI 0.35–0.77, data not shown).

Following a comprehensive evaluation of the currently available evidence and taking into account the benefits and possible harms associated with prostate cancer screening, it was decided against including a recommendation on prostate cancer [64]. Using MRI with targeted biopsy may reduce overdiagnosis, as reflected in the meaningful reduction of clinically insignificant prostate cancers while maintaining similar or even higher detection of clinically significant cancers. However, there were concerns related to current available evidence. For example, all but one of the studies were diagnostic accuracy studies, not conducted in a screening setting. Moreover, it is unclear whether the clinically significant cancers with MRI‐guided biopsy are the same as those detected with systematic biopsies due to grade shift [64] and the feasibility of MRI triage is limited by MRI capacity. There are currently several ongoing prostate cancer screening trials in Europe that can evaluate the results of multiple screening rounds, and the interval cancer rate [65, 66, 67].

Based on the currently available evidence, it is too early to recommend participation in prostate cancer screening. However, it is recommended to await the results of ongoing trials and incorporate them into a future update of the ECAC.

### 3.2 Presentation of the recommendation

The updated ECAC5 screening recommendation for individuals of the public is:

Take part in organised cancer screening programmes, as recommended in your country, for:

- Bowel cancer
- Breast cancer
- Cervical cancer
- Lung cancer

#### 3.2.1 Colorectal, breast and cervical cancer screening

##### 3.2.1.1 Equity

Participation rates tend to be lower among individuals with lower education levels, lower income, unemployment or immigrant status. This trend is also observed across all organised cancer screening programmes; however, this disparity is less pronounced compared to opportunistic screening [31, 68, 69, 70]. These findings suggest that while inequities in access to screening persist, organised programmes can help to reduce these disparities more effectively than opportunistic screening, thus ensuring universal health coverage by providing access to all [71, 72].

To promote equity in cancer screening, organised programmes should be implemented [73, 74]. Currently, although cancer screening is available in many European countries, not all programmes meet the standards for population‐based organised cancer screening. This shortfall hinders equitable access to cancer screening programmes. Barriers such as stigmatising views or cultural beliefs regarding cancer screening or diagnoses should be removed, as should barriers to access, such as providing information in different languages and improving clinic accessibility. This can be achieved, for example, by training the healthcare providers, inviting the entire eligible population and reaching out to hard‐to‐reach populations.

##### 3.2.1.2 Feasibility

The feasibility for individuals to follow this recommendation is facilitated by offering well‐organised cancer screening programmes, which are currently available for colorectal, breast and cervical cancer in most European countries [5]. Feasibility is further enhanced by the availability of home‐based self‐testing for colorectal cancer and, more recently, also HPV testing, which can be performed using either a self‐sample or one collected by a healthcare professional. The availability of self‐sampling methods, which may be more comfortable and convenient, can enhance participation for individuals so far not participating in regular screening, especially in remote areas or where there is limited access to healthcare providers [75]. For breast cancer screening, feasibility is associated with the availability of mammography facilities and the geographical location of the screening unit. Accessibility may be facilitated using mobile units. For all cancer screening programmes, it is crucial to ensure that individuals who receive a positive test result have timely access to high‐quality further diagnostic procedures and treatment options. The feasibility of these follow‐up tests depends on cost coverage. Those lacking resources or health insurance face barriers to access [76]. Taken together, the overall assessment is that participating in these three screening programmes is feasible.

##### 3.2.1.3 Acceptability

The acceptability of cancer screening is related to a variety of factors, which are not cancer‐specific but for cancer screening in general, including cultural norms, levels of trust in the health system, health literacy, the structure of the health system, the economic situation of the country and individual factors such as socio‐economic status [68, 70, 77, 78, 79, 80]. As cervical cancer screening includes the detection of sexually transmitted infections and usually requires gynaecological examination, acceptability is affected by the prevailing stigma associated with sexually transmitted infections and feelings of shame [38, 75]. Acceptability of cervical cancer screening may be enhanced by the introduction of self‐sampling, which is currently being implemented in several European countries, for example the Netherlands or Sweden. There is conflicting evidence on the acceptability of endoscopic screening, as the procedure is invasive and requires bowel preparation. In contrast, FIT screening is widely accepted across Europe, achieving high participation rates that reflect the acceptability of this noninvasive test, which can be performed at home [81]. Breast cancer screening, which has a long history with high uptake rates among eligible women in Europe, reflects the high acceptability of the screening. In conclusion, the acceptability of offered cancer screening tests is high in Europe.

#### 3.2.2 Lung cancer screening

As lung cancer is a new programme to be implemented and has not been described in a previous ECAC, equity, feasibility and acceptability are described separately.

##### 3.2.2.1 Equity

As lung cancer is more prevalent among individuals with = a lower socio‐economic background, it is anticipated that lung cancer screening will have a proportionally greater impact on these groups [82]. This, in turn, may help to improve equity by reducing inequalities in cancer mortality. LDCT screening has also been shown to have a greater impact on other common smoking‐related diseases in individuals from lower socio‐economic backgrounds [82]. Participation in screening programmes in general is lower in more deprived groups, prompting interventions to increase participation. In lung cancer screening, this is of particular importance because the incidence of the disease is much higher in these groups [83]. Research has shown that participation rates can be improved through tailored invitation methods [84]. Lung cancer screening may be more effective for women than for men. However, due to historical smoking patterns, more men than women currently qualify for screening. The gap between men and women is narrowing due to changes in smoking behaviour [85].

##### 3.2.2.2 Feasibility

At present, many countries do not have an organised lung cancer screening programme, so it is not possible for individuals to participate. However, it was decided for the ECAC5 to include a recommendation for LDCT screening to encourage countries to implement organised lung cancer screening. In light of the EU Council Recommendation for a stepwise implementation of lung cancer screening—ensuring gradual and appropriate planning, piloting and programme roll‐out—feasibility is anticipated to increase substantially, as several pilot and implementation studies are already underway. LDCT scanning is fast and completely painless. Accessibility may be facilitated using mobile units as a short distance to the screening unit can facilitate screening uptake, especially in those living in more deprived areas and those suffering from comorbidities, resulting in fewer transportation options. Stepwise implementation is needed to ensure sufficient capacity and resources for screening, work‐up and treatment [83].

##### 3.2.2.3 Acceptability

LDCT scanning is a suitable and highly acceptable procedure for the target population. The procedure is noninvasive and quick, with a maximum duration of 10 minutes for the screening appointment. Participants do not need to prepare and remain fully clothed. Waiting for the screening results can induce anxiety, like other screening programmes, but the effect is temporary [86]. Furthermore, preventing stigmatising the target population due to the direct relation of lung cancer and smoking can stimulate acceptability among the target population as well as the general public. Thereby, tailored multi‐channel communication can support informed decision‐making, especially in those with lower health literacy, women and current smokers.

#### 3.2.3 Co‐benefits for prevention of noncommunicable diseases other than cancer with similar risk factors and opportunities for health promotion

In addition to the direct benefits of screening, participation may also result in several co‐benefits. One such benefit may be the opportunity to also deliver lifestyle interventions, which may vary depending on the specific type of cancer [87, 88]. Lifestyle interventions that have been demonstrated to be effective in the prevention of colorectal cancer include a reduction in the consumption of processed and red meat and alcohol, and an increase in the intake of vegetables and dietary fibre [89, 90]. A healthy weight, physical activity and the avoidance of alcohol are also effective lifestyle interventions for the prevention of breast cancer. In addition to the lifestyle interventions, cervical cancer screening provides an opportunity to recommend HPV vaccination to children of attendees [8]. Lung cancer screening can also serve as an effective incentive for smoking cessation, with quit rates of 20–30% and it should therefore be coupled with the offer of smoking cessation advice, as well as quitting smoking interventions. Quitting smoking has been shown to reduce the risk of developing lung and other cancers and other serious conditions, including chronic lung diseases and cardiovascular diseases [91].

## 4 Recommendation for policymakers

Table 1 presents the European Code Against Cancer, 5th edition recommendations for policymakers on organised cancer screening programmes.

**Table 1:** European Code Against Cancer, 5th edition: recommendations for policymakers on organised cancer screening programmes. The table presents the recommendations for policymakers on the implementation of sustainable, organised cancer screening programmes.

Organised cancer screening programmes	
**Implement sustainable, organised screening programmes for colorectal (bowel), breast and cervical cancer:** *	
o	For colorectal cancer screening, implement quantitative faecal immunochemical test (FIT) every 2 years for individuals aged 50–74 years. Once‐only endoscopy may be considered as an alternative strategy within the same age range.
o	For breast cancer screening, implement digital mammography every 2 years for women** aged 50–69 years, and consider implementing it for women aged 45–49 years and 70–74 years. Other screening tools or additional examinations should be considered for women with high mammographic density.
o	For cervical cancer screening, implement HPV screening at intervals no shorter than 5 years for women** aged 30–65 years. Policies can be adapted according to vaccination status and screening history.
**Implement sustainable, organised screening programmes for lung cancer.** * **Implement low‐dose computed tomography every year (preferred) or every 2 years with integrated smoking cessation interventions for individuals identified as being at increased risk of lung cancer based on criteria of either age and history of smoking or locally validated multivariable risk models.**	

* The recommendations are subject to updates to reflect scientific and technological advances as specified in the European Guidelines for Cancer Screening and Diagnosis: https://cancer‐screening‐and‐care.jrc.ec.europa.eu;

** Includes people assigned female at birth who are eligible for this screening.

© 2026 International Agency for Research on Cancer / WHO. Used with permission.

References: • Council recommendation on strengthening prevention through early detection: a new EU approach on cancer screening replacing Council Recommendation 2003/878/EC. Brussels: European Commission; 2022. Available from: https://eur-lex.europa.eu/legalcontent/ [4]. • European Guidelines for Quality Assurance in Colorectal Cancer Screening and Diagnosis. https://op.europa.eu/en/publication-detail/-/publication/e1ef52d8-8786-4ac4-9f91-4da2261ee535 [94]. • International Agency for Research on Cancer. Handbook of cancer prevention. Colorectal cancer screening. Vol **17**. Lyon: IARC; 2019. http://publications.iarc.fr/573 [96]. • European guidelines breast cancer screening and diagnosis. https://cancer-screening-and-care.jrc.ec.europa.eu/en/ecibc/european-breast-cancer-guidelines [26]. • Karsa L, Dillner J, Suonio E, Tornberg S, Anttila A, Ronco G, et al. European guidelines for quality assurance in cervical cancer screening: second edition: supplements. 2015 https://doi.org/10.2875/859507 [95]. • WHO guideline for screening and treatment of cervical pre‐cancer lesions for cervical cancer prevention, second edition. 2021 Geneva: World Health Organization. https://www.who.int/publications/i/item/978924003082 [93]. • Baldwin DR, O’Dowd EL, Tietzova I, Kerpel‐Fronius A, Heuvelmans MA, Snoeckx A, et al. Developing a pan‐European technical standard for a comprehensive high‐quality lung cancer computed tomography screening programme: an ERS technical standard. *Eur Respir J*. 2023;**61**(6):2300128 [92].

### 4.1 Presentation of the recommendation for policymakers and key stakeholders

Policy recommendations in ECAC5 are based on the EU Council Recommendation [4, 97], European guidelines [26, 47, 92, 93, 94, 95, 98] and the three IARC Handbooks on cervical [31, 93], breast [26, 27] and colorectal cancer [31, 99]. We first provide a general recommendation on implementation of cancer screening followed by specific recommendations on test, age range and interval by cancer type.

#### 4.1.1 General recommendations for cancer screening

To reduce the cancer burden and minimise screening‐related harms and costs, equitable access to screening and timely, high‐quality follow‐up care must be ensured. The first policy action is to implement sustainable, well‐organised, population‐based screening programmes. Sustainability in the context of cancer screening can be defined as actively and effectively translating policies into practice to achieve a meaningful reduction in population‐level cancer burden. Sustainability requires consistent investment in coordination and quality assurance—both in screening and related health services—without delaying diagnosis and treatment for symptomatic patients. A well‐organised programme can be defined following the criteria for organised cancer screening of Zhang *et al*. [100]. Of the 16 essential criteria identified, particular emphasis is placed on the presence of a protocol or guideline outlining the target population, screening intervals, screening tests, referral pathways and management of positive cases. Additionally, a system must be in place to identify eligible individuals, along with a designated organisation or team responsible for implementing and/or coordinating the screening programme. Aligning with these criteria helps ensure that cancer screening is well‐organised, which can lead to accessible services for the entire eligible population and promote equitable access in line with the principles of Universal Health Coverage. Promoting equity in screening is essential. Informational materials should be tailored to the entire target population, with a focus on groups less likely to participate, such as migrants and those from lower socio‐economic backgrounds [68, 70]. Communication should be adjusted to the needs of the individuals; for instance, individuals with lower health literacy are more responsive to visually engaging messages or decision aids, such as web‐based tools, which can help support informed decision‐making [68, 69, 101]. Lastly, cancer screening should be free, to remove the financial barriers, especially for the more deprived populations. Specific policy actions are required for each cancer screening programme as outlined below.

Since cancer screening targets largely healthy populations, ensuring high‐quality services across the prevention and care continuum is essential. This requires strong quality assurance through standards, clinical and management guidelines, and digital systems like screening registries. These registries enable personal invitations, individual tracking, and comprehensive monitoring of procedures and outcomes, supporting programme evaluation and safety. Effective programme implementation depends on adequate resources, infrastructure, governance, legal frameworks, IT systems and trained personnel.

#### 4.1.2 Specific recommendations for colorectal cancer

For the policy recommendation on colorectal cancer screening, we used the IARC handbook and the European guidelines on colorectal cancer screening and diagnosis as reference [89, 92]. The RCTs on colorectal cancer screening generally included individuals aged 50–74, all demonstrating that the balance of harms and benefits favours screening. For individuals aged 45–49 years, the balance is less certain due to the lower prevalence of the disease. The rising incidence at younger age was discussed in the ECAC5 Working Group, but it was concluded that there is currently no justification to recommend a lower starting age [102]. Biennial FIT is still the recommended screening interval—supported by strong evidence—but in the future it is expected that screening intervals may be tailored to individual risk. A once‐only endoscopy screening is recommended, as existing trials were designed for a single screening and thus provide no evidence of the benefits of repetition [31].

The quantitative FIT allows for adjusting the positivity threshold to minimise false positives. It also enables countries to align with their available colonoscopy capacity. Since FIT‐based screening occurs without direct healthcare professional involvement, providing clear and easy‐to‐follow test instructions is essential.

#### 4.1.3 Specific recommendations for breast cancer

For the policy recommendation on breast cancer screening, we used the IARC handbook and the European guidelines on breast cancer screening and diagnosis as reference [26, 27]. The strength of the evidence on the reduction of breast cancer mortality is strong evidence for women aged 50–69 years, demonstrating that the balance of harms and benefits favours screening in this age group [26]. The evidence for women aged 45–49 years and 70–74 years was moderate. Following careful considerations, the consensus was to conditionally recommend screening for women aged 45–49 years and 70–74 years, only if capacity in the country allows. A similar discussion emerged on the optimal screening interval. In the absence of studies directly comparing biennial and triennial intervals, and considering local programme contexts, consensus supported a 2‐year interval [26].

Future screening may use risk‐based screening intervals. Risk stratification can identify high‐risk women—such as those with mammographic dense breasts—and offer them more intensive screening. It is suggested that women with high mammographic breast density at their first screening could be offered additional digital breast tomosynthesis [26]. The overall certainty of the evidence for this approach is still very low. Other screening tools or additional examinations should be considered for women with high mammographic density [26]. To optimise breast cancer screening, AI algorithms can be used for screen reading, potentially increasing the sensitivity and specificity of the screening test [103, 104, 105].

#### 4.1.4 Specific recommendations for cervical cancer

For the policy recommendation on cervical cancer screening, we used the IARC handbook and European guideline as reference [31, 95, 106]. For cervical cancer screening, HPV screening should be recommended for women aged 30–65 years. Policies can be adapted according to vaccination status and screening history. For younger women, aged 25–29 years, HPV testing may also have some advantages. The decision is a balanced one, considering the high prevalence of HPV at a young age, as well as the occurrence of cytological abnormalities, which are common but transient, and the relatively low incidence of CIN3+ [107]. It is strongly recommended that the screening interval is no shorter than 5 years.

Adequate triage of HPV‐positive women should be implemented, considering the underlying risk and HPV type for all age groups, especially in younger, unvaccinated individuals, considering the high prevalence of HPV and the possible obstetrical impact of overtreatment in this age segment [107]. Engaging the target population in cervical cancer screening follows a similar approach to colorectal and breast cancer screening. Providing self‐sampling kits has been shown to increase participation rates among nonresponders by offering a more convenient and accessible screening alternative [75].

#### 4.1.5 Specific recommendations for lung cancer

For the policy recommendation on lung cancer screening, we used guidelines from European medical societies [92]. For lung cancer screening, no specific starting age is recommended, as age should be integrated as part of the risk stratification. However, it is not recommended to start screening before the age of 50 or to continue beyond the age of 80. It is recommended that individuals undergo annual screening. In the absence of any abnormality suggestive of a further increased risk of lung cancer, as indicated by the preceding scan, biennial screening may be considered an option, although this is currently an ongoing area of research.

Risk‐based lung cancer screening is an important element that defines screening eligibility based on individuals' personal risk. Individuals at highest risk of lung cancer are more likely to maximise benefits of screening, while minimising potential harms caused by screening. The most optimal scenarios include high‐risk individuals based on age, a history of tobacco smoking (either current or previous), and other factors, some of which are related to smoking and others not [108]. Eligibility may be defined according to age and history of smoking alone (typically age, pack‐years and quit time duration) or by multivariable risk prediction models. Multivariable models include factors such as chronic obstructive pulmonary disease (COPD), a family history of lung cancer, personal history of cancer, body mass index and exposure to asbestos [109]. Thus, a clear protocol is recommended to define eligibility according to risk of developing lung cancer. There is some evidence that multivariable models lead to more efficient selection of participants. The risk threshold can be varied according to cost‐effectiveness calculations.

Accurate pulmonary nodule management is essential, preferably considering volume and growth of the solid part of nodules, as it has been demonstrated to be a reliable indicator of the probability of malignancy [110, 111]. This approach has led to low referral rates and subsequent test positives, while maintaining lung cancer mortality reductions. Separate cut‐offs are needed between existing and newly detected nodules, due to the increased probability of malignancy in the new nodules [111, 112]. Lung cancer screening should be accompanied by smoking cessation offers [91, 113, 114, 115].

#### 4.1.6 Feasibility and resources required

For all cancer screening programmes, infrastructure and resource capacity should be evaluated before implementation and monitored during and after implementation to ensure equal access to care for all individuals, either from the screening pathway or due to symptoms.

##### 4.1.6.1 Colorectal, breast and cervical cancer screening programmes

For colorectal cancer, implementing FIT‐based screening is feasible as it is an inexpensive test and requires limited resources for analyses. The primary constraint is the restricted availability of endoscopy resources, which are essential for the timely evaluation of individuals with FIT‐positive results. Nevertheless, the FIT positivity threshold can be adjusted to align with the availability of colonoscopy resources at the local level [116]. Endoscopy can be employed as the primary screening tool to implement strategies that combine the offer of endoscopy and FIT (offering a choice or adopting sequential strategies). As endoscopy capacity is often limited, the feasibility and accessibility of offering endoscopy as a primary screening tool (in particular colonoscopy) may be more challenging [4]. Breast cancer screening using mammography is feasible. It requires sufficient mammography machines and manpower to carry out the screening. Currently, some European countries are struggling to find and train qualified personnel to conduct screening and follow‐up examinations. As a result, the population‐based breast cancer screening programmes in Europe differ in terms of the target age group, the subsequent assessment and the associated costs [74]. Additionally, adequate resources must be available for subsequent assessment and treatment options [27]. Cervical cancer screening requires resources for sample collection, testing and follow‐up. HPV testing is more reliable, easier to interpret and requires less skilled laboratory personnel than cytology, addressing challenges in current programmes. To ensure acceptability, clinicians must provide respectful, culturally sensitive care. In some settings, female healthcare professionals can reduce barriers. Self‐sampling kits also empower women to screen in a private setting without clinical involvement [75].

##### 4.1.6.2 Lung cancer screening programme

For lung cancer screening, which is currently only implemented in a few countries across Europe, adequate preparation through carefully designed protocols that include quality assurance is critical, as a phased implementation is needed to ensure a high‐quality lung cancer screening programme. Stepwise implementation using evidence‐based screening standards and adequate access to diagnostics work‐up and treatment are needed to ensure a high‐quality lung cancer screening programme [92, 117, 118]. Implementation programmes/pilots/trials in Europe show that screening for lung cancer with LDCT is feasible. However, the quality of programmes differs across countries/healthcare systems [111, 119].

It is important to define the target population based on lung cancer risk *and* availability of resources. While countries have taken steps to implement this initiative, the lack of necessary resources is a significant barrier to progress [120]. Lung cancer screening must identify individuals with a smoking history, preferably by using patient records or an equivalent electronic database. Integrated smoking cessation should be feasible, as several studies have shown that smoking cessation rates are much higher within screening programmes [91, 115]. A clear protocol for (severe) incidental findings management is crucial to prevent unnecessary diagnosis and treatment procedures [47, 92]. Moreover, screening will lead to an increase in lung cancer diagnoses, which require follow‐up diagnostics and treatment. Sufficient healthcare resources should be available to handle this workload.

#### 4.1.7 Cost‐effectiveness of cancer screening programmes

Cost‐effectiveness analyses (CEAs) for colorectal, breast and cervical cancer screening have shown that organised cancer screening is cost‐effective [27, 31]. For colorectal cancer, FIT screening has been shown to be cost effective and even cost saving, as the removal of polyps and the shift to earlier stage cancers avoids costly treatment of (advanced) cancers, while the cost of the screening test itself is low [96]. Cost per quality‐adjusted life‐year (cost/QALY) ranged from cost saving to €6000 [121, 122]. FS has been shown to be cost saving as a result of the removal of adenomas interrupting their progression toward an invasive colorectal cancer and thus substantially reducing treatment costs. For breast cancer, biennial screening has been shown to be the most cost‐effective. Annual screening is effective, but the more intensive screening strategy leads to a large increase in costs and in higher frequency of side effects (unnecessary biopsies), resulting in less cost‐effective strategies [27]. Most CEAs showed costs/QALY of biennial mammography compared to no screening ranging between €5000 and €25 000 [123]. The use of artificial intelligence in interpreting screening mammograms has the potential to make breast cancer screening more cost‐effective [124]. Cervical cancer screening has shown to be cost‐effective, with cost/QALYs ranging between €2000 and €15 000 [125]. HPV testing compared to cytology testing has proven to be more cost‐effective, with recent studies suggesting that HPV testing can be cost saving [126]. For lung cancer screening, screening with LDCT is also considered to be cost‐effective, with cost/QALY ranging between €9000 and €85 000 [127]. The cost‐effectiveness of lung cancer screening is largely driven by the cost of CT screening and the cost of late‐stage cancer treatment in each country [128, 129]. Integrated smoking cessation can improve the cost effectiveness so that there is net monetary benefit [130, 131]. Thus, all four cancer screening programmes are generally regarded as cost‐effective. However, the cost per life‐year gained varies significantly, driven by factors such as the cost of primary screening tests, disease prevalence and cancer treatment expenses, among other variables.

## 5 Conclusions

Based on the available evidence and thorough discussion and consensus among the ECAC5 Working Group of experts, ECAC5 recommends individuals to take part in organised screening programmes for colorectal, breast, cervical and lung cancer, as recommended in their country. Correspondingly, European countries should aim to implement sustainable, organised screening programmes for these cancer types to promot their population in participating in screening. The Working Group decided against recommending prostate cancer screening although PSA screening was considered to be effective. When using systematic biopsy to assess all PSA positive screenees, the benefits of screening do not outweigh the harms. MRI with targeted biopsy significantly reduces the biopsy frequency and it may reduce overdiagnosis and thus harms of screening. However, when the recommendations were developed, there was insufficient evidence to determine whether the benefits of screening would be maintained with this approach.

To ensure that screening is effective and safe, policymakers must implement well‐organised programmes that guarantee equal access, sufficient participation rates, and high‐quality health services across the entire continuum of cancer prevention and care. A strong focus on quality assurance, including continuous monitoring and evaluation of the programme, is essential to maintain high standards and improve outcomes.

## Conflict of interest

The authors declare no conflict of interest. Where authors are identified as personnel of the International Agency for Research on Cancer/World Health Organization, the authors alone are responsible for the views expressed in this article and they do not necessarily represent the decisions, policy or views of the International Agency for Research on Cancer/World Health Organization.

## Author contributions

ET‐Z and IL‐V were responsible for writing the first version of the manuscript. All authors gave critical revisions on the intellectual content of the manuscript and approved the final manuscript.

## Supporting information


**Annex S1.** European Code Against Cancer, 5th edition. © 2026 International Agency for Research on Cancer / WHO. Used with permission.

**Annex S2.** Overview of PICOD questions for meta‐analysis on lung and prostate cancer screening.

**Annex S3.** Characteristics of reviews included in the review on lung cancer screening.

## Data Availability

The data that supports the findings of this study are available in Fig. 2 and Fig. 3 and the Supporting Information of this article.
