# Supplementary material for: European Code Against Cancer, 5th edition – organised cancer screening programmes
Source: Mol Oncol. 2026 Jan 16;20(1):134–53. doi: 10.1002/1878-0261.70197 (PMC12809467; doi:10.1002/1878-0261.70197)
Supplement: Supplementary file 2 — Annex S2. Overview of PICOD questions for meta‐analysis on lung and prostate cancer screening. [file MOL2-20-134-s003.pdf]

Annex S2 – Overview of PICOD questions

Effectiveness for lung cancer screening

**Population**	Adults at high risk of developing lung cancer.
**Intervention**	Volumetric-based and diameter-based screening.
**Comparison**	LDCT versus no screening or chest x-ray.
**Outcomes**	Disease-specific mortality; all-cause mortality; rate of advanced lung cancer; lung cancer incidence; smoking behaviour; incidental findings (benefits); harms.
**Design**	i. Overview of systematic reviews<br>ii. Systematic review of randomized controlled trials.

Effectiveness for prostate cancer screening

**Question**	What is the balance of benefits and harms of using Magnetic Resonance Imaging (MRI) as a triage test following a positive Prostate-specific antigen (PSA) test compared with usual care / PSA only followed by systematic biopsy for prostate cancer detection?
**Population**	Male population aged 50 to 69 years old with or without abnormal prostate cancer screening (PSA or digital exam) and no prior prostate biopsy.
**Intervention**	MRI followed by targeted biopsy in case of MRI findings suggestive of malignancy.
**Comparison**	Comparator transrectal ultrasound guided biopsy (TRUS-GB) or transperineally template biopsy (TPT-B).
**Outcomes**	Disease-specific mortality; all-cause mortality; adverse events; biopsy frequency; clinically significant prostate cancer; clinically insignificant prostate cancer.
**Design**	Systematic review of randomized controlled trials.
