# Supplementary material for: European Code Against Cancer, 5th edition – organised cancer screening programmes
Source: Mol Oncol. 2026 Jan 16;20(1):134–53. doi: 10.1002/1878-0261.70197 (PMC12809467; doi:10.1002/1878-0261.70197)
Supplement: Supplementary file 3 — Annex S3. Characteristics of reviews included in the review on lung cancer screening. [file MOL2-20-134-s002.pdf]

Supplementary 3 – Characteristics of reviews included in the review and assessment of the evidence

	Review basic data and methods				Population		Interventions features			Outcomes
Study ID	Searched Databases (restriction; Date of last search)	Eligibility Criteria	Risk of bias / Certainty of evidence assessments	Nº and design of included studies	Age	Smoking history	LDCT parameters	Comparator	Frequency of scanning	Outcomes reported in the review
**Prioritized reviews**										
Bonney 2022 [132]	CENTRAL, MEDLINE and Embase , Cochrane Lung Cancer Group Trial Register, conference proceedings, trial registries, reference lists and authors contact (None; July 31, 2021)	RCTs, with adults without previous diagnosis of lung cancer, asymptomatic, assessing LDCT (volumetric CT dose index of ≤3) compared to no screening or any non-LDCT intervention	Cochrane RoB tool / GRADE approach	11 RCTs	Most trials included adults aged 40 years or older	Most included trials had an entry requirement of ≥ 20 pack-year smoking history	From 80kV to 140kV and 20mA to 100mA	No screening (seven RCTs); chest x-ray (four RCTs)	Annual (nine RCTs), biennial (one RCT) and incrementing intervals (one RCT)	Lung cancer mortality, all-cause mortality, cancer incidence, stage at diagnosis, histology, false positives, smoking behavior, use of biomarkers, response rate, adherence to screening, contamination, interval of lung cancer, false negatives, cost, medication implications. Harms due to: clinical investigations in false positives, any complications arising from tests (including death), radiation exposure, incidental findings, nodule management, HRQoL (including psychological impact)

Jonas 2021 [133]	Cochrane and MEDLINE, trial registries and reference lists (English language; from January 1, 2012 to May 28, 2019)	Studies of good or fair quality as per the USPSTF criteria, published in or after 2001, conducted in countries of “Very High” HDI*, with a sample size ≥ 500 (harms due to treatment after LDCT) or ≥1000 (other outcomes) and on LDCT in asymptomatic adults. For the question on harms of treatment, participants with Stage I lung cancer	USPSTF for RCTs and ROBINS-I / Method developed for the USPSTF (and the EPC program)	7 RCTs and 38 observational studies	Most studies included people with mean or median ages over 60 years old.	NR	From 120 to 180 kV, 20mA to 80 mA	A comparison was not required	Annual in most included trials	Lung cancer mortality, all-cause mortality, lung cancer incidence (all stages), distribution of lung cancer types and stages, smoking cessation, sensitivity, specificity, predictive value, false positives, overdiagnosis, unnecessary treatment. Harms due to: Incidental findings, workup (biopsy), radiation dose, clinical investigations, biopsies, treatment (including mortality, infection, bleeding, bronchopleural fistula, and respiratory failure), HRQoL or functional status (including psychological impact)
Passiglia 2021 [134]	CENTRAL, MEDLINE and Embase, ClinicalTrial.gov up (None; Date: up to February 13, 2020)	RCTs comparing LDCT to either no screening or chest x-ray in a high-risk population with a cigarette smoking history of at least 15 pack-years, including former smokers who had quit within the previous 15 years	Cochrane RoB tool / GRADE approach	9 RCTs	Most trials included adults aged 50-75 years old	Subjects with a cigarette smoking history	NR	Chest x-ray (three RCTs) or usual care (six RCTs)	Annual for chest x-ray	Lung cancer mortality, all-cause mortality, early stage and late stage incidences, resectability rate, overdiagnosis
**Non-prioritized reviews**										
Agrawal 2022 [135]	MEDLINE, Scopus, and CENTRAL, reference lists (None; up to January 2020)	RCTs published in English with adults without history of lung cancer who received LDCT for screening of lung cancer, compared to other form of screening or standard care	Cochrane RoB tool /None	9 RCTs	People aged from 45-75 years	From 20 to 30 pack-years of smoking history	NR	Chest x-ray or no screening	Annual/biennial	Lung cancer mortality, all-cause mortality and lung cancer detection

		reporting all-cause mortality,lung cancer mortality and incidence rate								
Chen 2022 [136]	MEDLINE, EMBASE, and CENTRAL, reference lists (None; up to January 2015)	RCTs with adults without history of lung cancer assessing LDCT, chest x-ray, sputum analysis or usual care for lung cancer screening reporting lung cancer mortality or all-cause mortality with a follow-up of at least 5 years	Cochrane RoB 2 tool / None	11 RCTs	Mean or median age ranged from 55 to 63 years	Mean or median pack-years ranged from 20 to 54.	NR	Chest x-ray, sputum analysis or no screening (with network meta-analysis)	NR	Lung cancer mortality, all-cause mortality, stage at diagnosis, false positive rate, quality of life, smoking cessation, deaths after invasive procedures
Hoffman 2020 [137]	MEDLINE, Google Scholar, Web of Science and CENTRAL, Clinicaltrials.gov, reference lists (English; published from January 2011 until April 2020)	RCTs on LDCT that reported lung cancer and/or overall mortality data	Cochrane RoB tool / GRADE approach	9 RCTs	Mean or median age was around 60	Mean and median pack-years of smoking was usually about 40 or more	NR	Chest x-ray or no screening	Annual/ biennial	Lung cancer mortality, all-cause mortality, detection of stage I lung cancer
Huang 2019 [138]		RCTs comparing LDCT to any other type of lung cancer screening in asymptomatic adults reporting lung cancer mortality, all-cause mortality, early detection (stage I) rates, death and major complications after invasive procedures (30–60 days post invasive procedures	Cochrane RoB 2 tool / None	9 RCTs	Mean or median age ranged from 57 to 64 years	Mean and median pack-years of smoking ranged from about 10 to 48	NR	Chest x-ray or no screening	Annual/ biennial	Lung cancer mortality, all-cause mortality, early stage cancer detection, deaths after invasive procedures, major complications after invasive procedures

Hunger 2021 [139]	MEDLINE, Embase, and CENTRAL, reference lists (None; up to April 2020)	RCTs published in English and German that compared LDCT with no screening or chest x-ray reporting benefits and/or harms of LDCT and covered participant selection to LDCT screening, lung cancer diagnosis, and follow-up.	Cochrane RoB tool / None	10 RCTs	People aged from 49 to 75 years	Studies included participants with a smoking history of ≥ 20 pack-year	From 80 to 140 kV and 20 to 100mA	Chest x-ray or no screening	Annual/ biennial	Lung cancer mortality, all-cause mortality, lung cancer incidence (including stage and histology), smoking behaviour radiation exposure, invasive procedures, false-positives, overdiagnosis, and HRQoL (including psychosocial impact)
Mazzone 2018 [140]	MEDLINE, Embase, and CENTRAL, reference lists (English; up to August 2017)	RCTs, systematic reviews and observational studies with asymptomatic adults with no history of lung cancer that compared LDCT with no screening, sputum analysis or chest x-ray	Cochrane RoB tool / GRADE approach	7 RCTs	Mean or median age ranged from 56 to 67 years	Median pack-years ranged from 30 to 56.	NR	Chest x-ray, sputum analysis or no screening	Annual/ biennial	Lung cancer mortality, lung cancer incidence (including stage distribution), smoking cessation, complications from biopsies of detected lesions (including death), surgery for benign disease, HRQoL (including distress, anxiety, depression), overdiagnosis, cost-effectiveness
Mazzone 2021 [141]	MEDLINE, Embase, and CENTRAL, reference lists (English; up to January 2020)	RCTs, systematic reviews and observational studies with asymptomatic adults with no history of lung cancer that compared LDCT with no screening, sputum analysis or chest x-ray	Cochrane RoB tool / GRADE approach	8 RCTs	People aged from 50 to 75 years	Most studies included participants with a smoking history of ≥ 15 pack-year	NR	Chest x-ray, sputum analysis or no screening	Annual/ biennial	Lung cancer mortality, lung cancer incidence (including stage distribution), smoking cessation, complications from biopsies of detected lesions (including death), surgery for benign disease, HRQoL (including distress, anxiety, depression), overdiagnosis, cost-effectiveness

Snowsill 2018 [142]	MEDLINE. EMBASE, PsycINFO, Web of Science, CENTRAL and CINAHL, Clinicaltrials.gov, WHOICTRP, EU clinical trials, ISCRTN, consultation with experts, reference lists (English, up to January 2017)	RCTs in adults comparing LDCT to usual care (no screening) or other imaging technology screening programmes (such as chest x-ray)	Cochrane RoB tool / None	6 RCTs	People aged from > 49 to 76 years	Studies included participants with a smoking history of ≥ 15 pack-year	From 80kV to 140kV and 20mA to 50mA	Chest x-ray, sputum analysis or no screening (with network meta-analysis)	Annual/ biennial	Lung cancer mortality, all-cause mortality, cancer incidence (including stage distribution), smoking behaviour, follow-up investigations, surgical treatment, surgical resection, HRQoL, adherence rate to screening, diagnostic accuracy outcomes , overdiagnosis, complications from invasive procedures, radiation dose and radiation-related outcomes, adverse psychological impact.
Tang 2019 [143]	MEDLINE, Web of Science, CENTRAL, ScienceDirect, China National Knowledge Infrastructure, and Chinese biomedical Database, reference lists, conference proceedings (None; up to February 26, 2019)	RCTs on LDCT versus X-ray or usual care screening for lung cancer	Critical Appraisal Skills Programme (CASP) tool / None	9 RCTs	People aged from > 49 to 75 years	Studies included participants with a smoking history of ≥ 15 pack-year	NR	Chest x-ray, or no screening	Annual/ biennial	Lung cancer mortality, all-cause mortality, cancer incidence, stage I incidence
Yang 2019 [144]	MEDLINE ,Embase, PsycINFO, Web of Science, CENTRAL and CINAHL, reference lists, consultation with experts (None; up to to January 2017)	RCTs in adults comparing LDCT to usual care (no screening) or other imaging technology screening programmes (such as chest x-ray)	Cochrane RoB tool / None	6 RCTs	People aged from > 49 to 74 years	NR	NR	Chest x-ray, sputum analysis or no screening (with network meta-analysis)	Annual/ biennial	Lung cancer mortality, all-cause mortality

Initial Certainty	Certainty assessment											Effect		Updated Certainty
	Nº of studies (participants)	Study design	Risk of bias	Inconsistency	Indirectness	Imprecision	Publication bias	Magnitude of effects	Dose response	Residual confounding	Consistency across populations & studies	Relative (95% confidence interval)	Absolute (95% confidence interval)	
**Disease-specific mortality: Follow-up: 6 years to 10 years from randomization**														
High	8 (91,122 participants)	RCT	Not serious	Not serious	Not serious	Not serious	Not detected	No	No	No	No	RR 0.79 (0.72 to 0.87)	4 fewer per 1,000 people screened (3 fewer to 6 fewer)	⊕⊕⊕⊕ High
**All-cause mortality: Follow-up: 6 years to 10 years from randomization**														
High	8 (91,107 participants)	RCT	Not serious	Not serious	Not serious	Not serious	Not detected	No	No	No	No	RR 0.95 (0.91 to 0.99)	4 fewer per 1,000 people screened (1 fewer to 8 fewer)	⊕⊕⊕⊕ High
**Rate of advanced cancer: Follow-up: 3-12.3 years**														
High	9 (8,641 participants)	RCT	Serious[a]	Not serious	Not serious	Not serious	Not detected	No	No	No	No	RR 0.75 (0.68 to 0.83)	5 fewer per 1,000 (6 to 3 fewer)	⊕⊕⊕◯ Moderate
**Cancer incidence: Follow-up: 7 years from randomization**														
High	8 (8,528 participants)	RCT	Serious[b]	Not serious	Not serious	Not serious	Not detected	No	No	No	No	RR 1.17, (1.02 to 1.33)	6 more per 1,000 (1 to 12 more)	⊕⊕⊕◯ Moderate

**Smoking behaviour: Assessed by the number of participants who quit smoking: Follow-up: 4 years after randomization**														
High	1 (2,447 participants)	RCT	Serious[c]	Not serious	Not serious	Serious[d]	Not detected	No	No	No	No	RR 1.17 (0.99 to 1.37)	31 more per 1,000 (2 less to 67 more)	⊕⊕◯◯ Low
**Incidental findings (benefits)**														
No trial evidence was available for this outcome														
